# Resveratrol Prevents Diabetic Cardiomyopathy by Increasing Nrf2 Expression and Transcriptional Activity

**DOI:** 10.1155/2018/2150218

**Published:** 2018-03-12

**Authors:** Guan Wang, Xianjin Song, Lei Zhao, Zhibo Li, Bing Liu

**Affiliations:** Department of Cardiology, The Second Hospital of Jilin University, Jilin University, Changchun 100032, China

## Abstract

**Objective:**

This study investigated if resveratrol ameliorates diabetic cardiomyopathy by targeting associated oxidative stress mechanisms.

**Method:**

Type 1 diabetes mellitus (DM) in FVB mice was induced by several intraperitoneal injections of a low dose of streptozotocin. Hyperglycemic and age-matched control mice were given resveratrol (10 mg/kg per day) for 1 month and subsequently monitored for an additional 6 months. Mice were assigned to four groups: control, resveratrol, DM, and DM/resveratrol. Cardiac function and blood pressure were assessed at 1, 3, and 6 months after DM induction. Oxidative damage and cardiac fibrosis were analyzed by histopathology, real-time PCR, and Western blot.

**Result:**

Mice in the DM group exhibited increased blood glucose levels, cardiac dysfunction, and high blood pressure at 1, 3, and 6 months after DM induction. Resveratrol did not significantly affect blood glucose levels and blood pressure; however, resveratrol attenuated cardiac dysfunction and hypertrophy in DM mice. Resveratrol also reduced DM-induced fibrosis. In addition, DM mice hearts exhibited increased oxidative damage, as evidenced by elevated accumulation of 3-nitrotyrosine and 4-hydroxynonenal, which were both attenuated by resveratrol. Mechanistically, resveratrol increased NFE2-related factor 2 (Nrf2) expression and transcriptional activity, as well as Nrf2's downstream antioxidative targets.

**Conclusion:**

We demonstrated that resveratrol prevents DM-induced cardiomyopathy, in part, by increasing Nrf2 expression and transcriptional activity.

## 1. Introduction

Diabetes mellitus (DM) is a well-documented risk factor for cardiovascular diseases (CVD), including cardiomyopathy. Previous studies have shown that patients with type 1 and type 2 diabetes exhibit both diastolic and systolic dysfunction, left ventricular (LV) hypertrophy, and cardiac fibrosis [1]. Diabetic cardiomyopathy (DCM) was initially defined in 1972 in a group of diabetic patients with congestive heart failure [2, 3]. Interestingly, these patients had no risk factors for heart failure or coronary artery disease but suffered from cardiomyopathies [2, 3]. At present, the incidence of heart failure due to diabetic myocardial damage is approximately 30% [4]. DCM pathogenesis is complex and may be related to a number of factors including high blood glucose levels, inflammation, oxidative stress, vascular endothelial injury, microangiopathy, cardiomyocyte hypertrophy, and fibrosis. Among these factors, oxidative stress is believed to play a key role in DCM pathogenesis [5–7].

Accumulating evidence indicates that prevention of the pathogenesis and progression of cardiac muscle disorders in diabetic patients cannot be achieved only through decreasing blood pressure, glucose levels, or lipid levels or by blocking the renin-angiotensin system [8]. Hence, more effective treatments to prevent the initiation and progression of cardiomyopathy are necessary to reduce diabetes-associated mortality.

It has been well documented that one of the major mechanisms underlying DCM pathogenesis is vascular damage, which is the consequence of chronic exposure to hyperglycemia and increased production of reactive oxygen species (ROS) [9]. Increased protein kinase C (PKC) activity and polyol and hexosamine flux [10], as well as potentiated formation of advanced glycation end-products (AGEs), also contribute to vascular damage. These signaling molecules converge on multiple inflammatory pathways, one of which is the nuclear factor-*κ*B (NF-*κ*B) signaling pathway. NF-kB signaling, once activated, increases expression of proinflammatory genes and secretion of proinflammatory factors such as cytokines [11].

The transcription factor NFE2-related factor 2 (Nrf2), which belongs to the cap “n” collar family, is an important mediator of detoxification and redox status [12]. At the basal level, Nrf2 is localized in the cytosol where it binds to its inhibitor kelch-like ECH-associated protein 1 (KEAP1). Following external assaults, such as electrophilic chemicals and oxidative stress, Nrf2 is released from KEAP1 and relocates to the nucleus where it binds to antioxidant-responsive sequences on gene promoters. This binding results in transcriptional activation of antioxidant enzymes, including *γ*-glutamylcysteine synthetase, glutathione S-transferase, heme oxygenase-1 (HO-1), catalase (CAT), NADPH, superoxide dismutase (SOD), and quinone oxidoreductase (NQO1), to antagonize oxidative stress-induced tissue inflammation and injury [13, 14].

Resveratrol is a natural polyphenol present in a variety of agricultural products such as red wine, grapes, and peanuts [15]. Since the discovery of resveratrol in 1940, it has been valued by the medical community because of its biological properties, which include antioxidant and anti-inflammatory effects, regulation of vasodilation, antiplatelet aggregation, and inhibition of smooth muscle cell proliferation. Resveratrol has also been shown to prevent and treat heart disease and atherosclerosis [16–18]. In addition, resveratrol can promote cardiac autophagy in DCM and during ischemia-reperfusion or hypoxia-reoxygenation [19]. However, it remains unclear if resveratrol can regulate Nrf2 signaling to mitigate oxidative stress in DCM.

We hypothesized that resveratrol protects against DCM by suppressing oxidative stress. The present study aimed to investigate the effects of resveratrol on cardiac function via the Nrf2 pathway in a mouse model of DM.

## 2. Materials and Methods

### 2.1. Animals

FVB male mice aged 8–10 weeks were purchased from the Beijing Wei Tong Lihua Experimental Animal Technical Co., Ltd., and housed under conditions of controlled light (0600–1800 h) and temperature (22 ± 2°C) with food and water available ad libitum. The Institutional Animal Care and Use Committee of Jilin University approved all experimental procedures. All experimental protocols were compliant with the Guide for the Care and Use of Laboratory Animals published by the US National Institutes of Health (NIH Publication Number 85-23, revised 1996).

To induce type 1 diabetes, mice were intraperitoneally given multiple low doses of streptozotocin (MLD-STZ) (Sigma-Aldrich, USA) at 40 mg/kg body weight per day for five consecutive days. Age-matched control mice received injections of sodium citrate buffer at the same volume. Five days after the last MLD-STZ injection, mice that had a blood glucose level ≥ 12 mmol/dl were designated as having diabetes, as previously described [20]. Resveratrol was given orally at 10 mg/kg mg per day for 1 month. After another month, a subset of mice was used for experimentation; the remaining mice were maintained for another 2 or 5 months (corresponding to 3 or 6 months after resveratrol treatment) and then were used for experimentation. Mice were randomly assigned to four groups (*n* = 18 per group): control, resveratrol, DM, and resveratrol plus DM (DM/resveratrol).

### 2.2. Noninvasive Blood Pressure Measurement

Blood pressure (BP) was determined by tail-cuff manometry using a CODATM noninvasive BP monitoring system. Briefly, unanesthetized mice were restrained in a plastic tube, which was placed on a heating pad to maintain body temperature and to ensure sufficient blood flow to the tails. Occlusion and volume-pressure recording cuffs were placed over the tail. Each mouse was permitted to adapt to the restrainer for 5 min before BP measurement. BP was measured for 10 acclimation cycles followed by 20 measurement cycles. Each mouse underwent three days of training before formal BP and heart rate measurements were acquired.

### 2.3. Echocardiography

Mice were anesthetized with avertin and placed on a warming pad, and cardiac function was evaluated using a two-dimensional M-mode Vevo 770 (VisualSonics, Canada). Chest hair was removed. Parasternal long-axis and short-axis views were used to obtain cardiac functional indices, including ejection fraction (EF), fractional shortening (FS), left ventricular (LV) dimensions, and wall thicknesses. The final data represented the average measurements of 10 cardiac cycles.

### 2.4. Histopathology

Mouse hearts were collected and fixed in 10% buffered formalin overnight. Hearts were then prepared using a standard procedure for tissue sectioning at 5 *μ*m thickness. Tissue sections were incubated in 1x target antigen retrieval solution (Dako, USA) in a microwave oven for 15 min at 98°C. Thereafter, tissue sections were treated with 3% hydrogen peroxide for 15 min at room temperature, followed by incubation in 5% bovine serum albumin (Sigma-Aldrich, USA) for 30 min. Cardiac fibrosis was evaluated with 0.1% Sirius-red F3BA and 0.25% Fast green FCF, as described previously [21].

### 2.5. Real-Time PCR

Total RNA was purified using the TRIzol Reagent (Invitrogen, Carlsbad, CA, USA). RNA purity and concentration were measured using a Nanodrop ND-1000 spectrophotometer. The first-strand complimentary DNA (cDNA) was generated from the total RNA with a RNA PCR kit (Roche, USA) according to the manufacturer's protocol. Reverse transcription (RT) was carried out with 1 *μ*g of total RNA in a reaction mixture in a final volume of 20 *μ*L. Reaction conditions for RT were 42°C for 50 min and 95°C for 5 min.

Primers for real-time PCR (qPCR) were purchased from Huada (Beijing, China) and their sequences are detailed below: connective tissue growth factor (CTGF): forward, 5′-GAGGAGTGCGTGTGTGACGA-3′; reverse, 5′-GGACCAGGCAGTTGGCTCTA-3′; heme oxygenase-1 (HO-1): forward, 5′-AGCCCCACCAAGTTCAAACA-3′; reverse, 5′-TGCCAACAGGAAGCTGAGAG-3′; metallothionein (MT): forward, 5′-CCATGTCGGGCAAGTGCG-3′; reverse, 5′-GCTGCTCAGTTTCAGTGA-3′; superoxide dismutase-1 (SOD1): forward, 5′-GGTGGGCCAAAGGATGAAGAG-3′; reverse, 5′-CCACAAGCCAAACGACTTCC-3′; superoxide dismutase-2 (SOD2): forward, 5′-TTCTGGACAAACCTCAGCCCTA-3′; reverse, 5′-AACCTGAGCCTTGGACACCA-3′; NAD(P)H quinone dehydrogenase-1 (NQO-1): forward, 5′-AGGACCCTTCCGGAGTAAGAA-3′; reverse, 5′-GTCAGGGAAGCCTGGAAAGA-3; *β*-actin: forward, 5′-CTTCCAGCCTTCCTTCCTGG-3′; reverse, 5′-TTCTGCATCCTGTCGGCAAT-3′. qPCR was performed in a 20 *μ*L reaction buffer with the ABI 7300 real-time qPCR system. Fluorescent intensity of each sample was determined at each temperature to monitor the amplification of the gene of interest. Comparative cycle time (CT) was utilized to determine the fold differences between samples [22].

### 2.6. Western Blot

Whole heart lysates were purified from homogenized heart tissues in RIPA buffer (Santa Cruz, USA) and sonicated. Protein concentrations were determined using the Bio-Rad DCTM protein assay. Protein samples were subjected to 10% SDS-PAGE followed by transfer to a nitrocellulose membrane (Bio-Rad, Hercules, CA, USA). The membrane was blocked with a 5% nonfat dried milk for 1 h, followed by an overnight incubation at 4°C with the following antibodies (all from Abcam, USA): CTGF at 1 : 500 dilution, 3-nitrotyrosine (3-NT) at 1 : 500 dilution, 4-hydroxy-2-nonenal (4-HNE) at 1 : 500 dilution, Nrf2 at 1 : 500 dilution, HO-1 at 1 : 500 dilution, NQO-1 at 1 : 500 dilution, MT at 1 : 500 dilution, SOD1 at 1 : 500 dilution, SOD2 at 1 : 500 dilution, and glyceraldehyde phosphate dehydrogenase (GAPDH) at 1 : 500 dilution. The membranes were washed with Tris-buffered saline (pH 7.2) containing 0.05% Tween 20 and incubated with appropriate secondary antibodies for 1 h at room temperature. Protein bands were visualized with an enhanced chemiluminescence kit (Thermo Scientific, Rockford, IL, USA) [21].

### 2.7. Statistical Analysis

Data are presented as means ± standard deviation (SD) or standard error of the mean (SEM) as indicated. Positive staining was identified with Image Pro Plus 6.0 software and protein bands were quantified with Image Quant 5.2. One- or two-way ANOVA followed by post hoc pairwise repetitive comparisons using Tukey's test was used to perform statistical analysis between groups with SAS 9.1 software (SAS, USA). *p* < 0.05 was considered statistically significant.

## 3. Results

### 3.1. Effects of Resveratrol on Body Weight and Blood Glucose Levels in Control and Diabetic Mice

**Figure 1:**
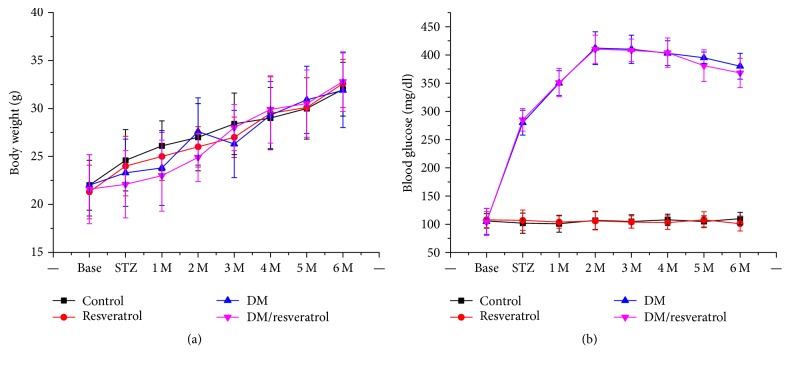
*Effects of resveratrol on body weight and glucose levels in mice from control, resveratrol, DM, and DM/resveratrol groups*. After induction of diabetes, body weight (a) and blood glucose levels (b) of mice were monitored monthly.

First, we measured body weight and nonfasting blood glucose levels in mice from four groups, control, resveratrol, DM, and DM/resveratrol, during and after resveratrol treatment (from 2 weeks to 6 months). We did not observe any significant difference in body weight among the groups (Figure 1). However, the DM and DM/resveratrol groups had significantly higher blood glucose levels compared to the other two groups, suggesting successful induction of DM. No significant difference in blood glucose levels was observed between the DM and DM/resveratrol groups (Figure 1).

### 3.2. Resveratrol Attenuates Diabetes-Related Cardiac Dysfunction and Hypertrophy

Next, we compared BP and HR between different groups of unanesthetized mice. As shown in Table 1, there was no difference in HR among the four groups; however, both systolic BP (SBP) and diastolic BP (DBP) gradually increased from 1 to 6 months in the DM group, which was significantly diminished by resveratrol treatment (Table 1).

**Figure 2:**
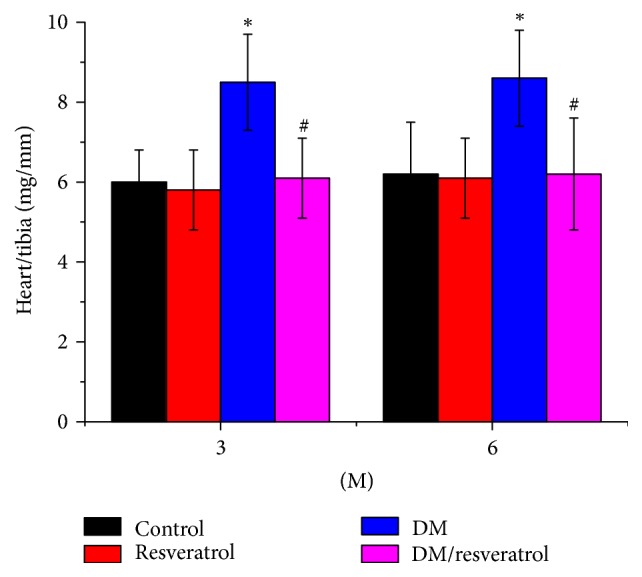
*Diabetes-induced cardiac hypertrophy*. At both 3 and 6 months after induction of diabetes, the ratio of heart weight to tibia length was calculated. Data are presented as means ± SD (*n* = 6 per group). ^*∗*^*p* < 0.05 versus control; ^#^*p* < 0.05 versus DM. DM: diabetes; 3 M or 6 M: 3 months or 6 months of diabetes.

Similarly, HR in the anesthetized mice was not significantly different between the groups (Tables 2, 3, and 4). The DM group exhibited gradual increases in IVS and LVPW and decreases in EF and FS from 1 to 6 months, indicative of cardiac dysfunction. These cardiac functional alterations were diminished by resveratrol. Consistent with the cardiac functional data, the DM group showed a progressive increase in the heart/tibia ratio (Figure 2, ^*∗*^*p* < 0.05 versus control), indicating an increase in heart mass and cardiac hypertrophy. However, this increase was significantly suppressed by resveratrol treatment (Figure 2, ^#^*p* < 0.05 versus DM). Taken together, these findings demonstrate that resveratrol protects against DM-related cardiomyopathy.

### 3.3. Resveratrol Prevents DM-Related Oxidative Stress and Cardiac Fibrosis

**Figure 3:**
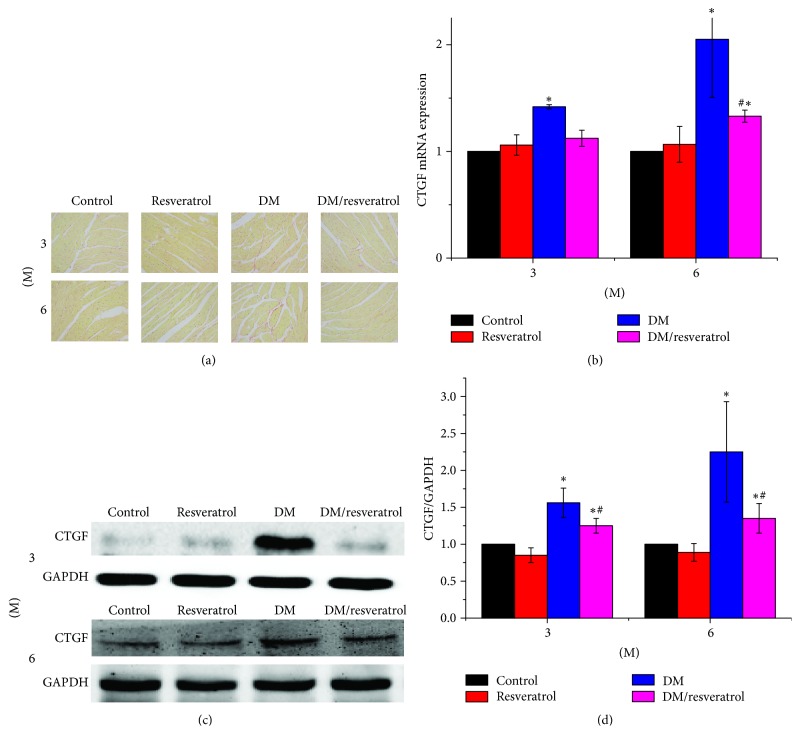
*Resveratrol prevents diabetes-related cardiac remodeling.* Heart sections were subjected to Sirius-red staining to measure collagen accumulation (a). CTGF expression was measured by quantitative PCR (b) and Western blot (c, d). Data are presented as means ± SD (*n* = 6 per group). ^*∗*^*p* < 0.05 versus control; ^#^*p* < 0.05 versus DM group. Bar = 100 *μ*m.

**Figure 4:**
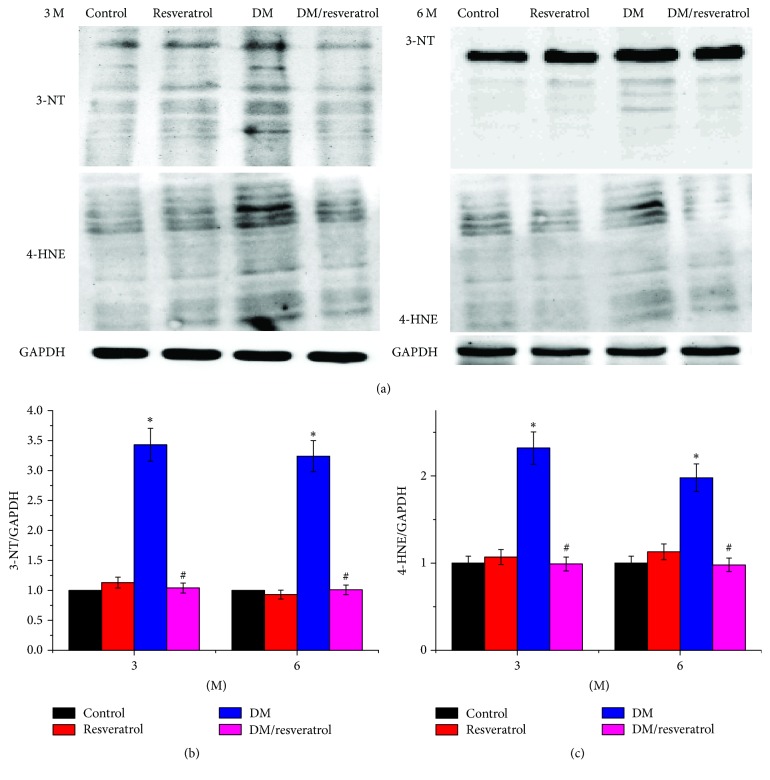
*Resveratrol attenuates diabetes-related oxidative stress*. Western blots were used to measure 3-NT (a, b) and 4-HNE (a, c) expression. Data are presented as means ± SD (*n* = 6 per group). ^*∗*^*p* < 0.05 versus control; ^#^*p* < 0.05 versus DM group. Bar = 100 *μ*m.

We next assessed fibrosis in DM hearts by Sirius-red staining of collagen (Figure 3(a)). Mice in the DM group had significant collagen accumulation, which was primarily detected in interstitial tissues but also observed in the perivascular area. Collagen accumulation increased with disease progression from 3 to 6 months. Resveratrol treatment attenuated the DM-related cardiac fibrosis. We evaluated the damage of mouse cardiomyocytes by histological hematoxylin-eosin (HE) staining (Supplementary Figure (available here)). The results showed that the myocardial cells of DM mice were disordered, with hyperemia in the small blood vessels. However, no significant damage was observed in cardiomyocytes in resveratrol treatment group. The increased cardiac fibrosis in DM hearts was further verified by elevated CTGF expression at both mRNA and protein levels (Figure 3(b), ^*∗*^*p* < 0.05 versus control). Both DM-related fibrosis and CTGF expression were reduced in the DM/resveratrol group compared to the DM group (Figures 3(b)–3(d), ^#^*p* < 0.05 versus DM). Next, we evaluated DM-related cardiac oxidative damage by measuring 3-NT accumulation as an indicator of nitrosative damage and 4-HNE accumulation as an indicator of lipid peroxidation at 3 and 6 months (Figure 4). As expected, DM significantly induced the levels of 3-NT and 4-HNE (^*∗*^*p* < 0.05 versus control), which were reversed by resveratrol treatment (^#^*p* < 0.05 versus DM). Taken together, we conclude that resveratrol suppresses cardiac fibrosis and reduces oxidative damage in DM.

### 3.4. Resveratrol-Mediated Cardiac Protection Correlates with Increased Nrf2 Activity

**Figure 5:**
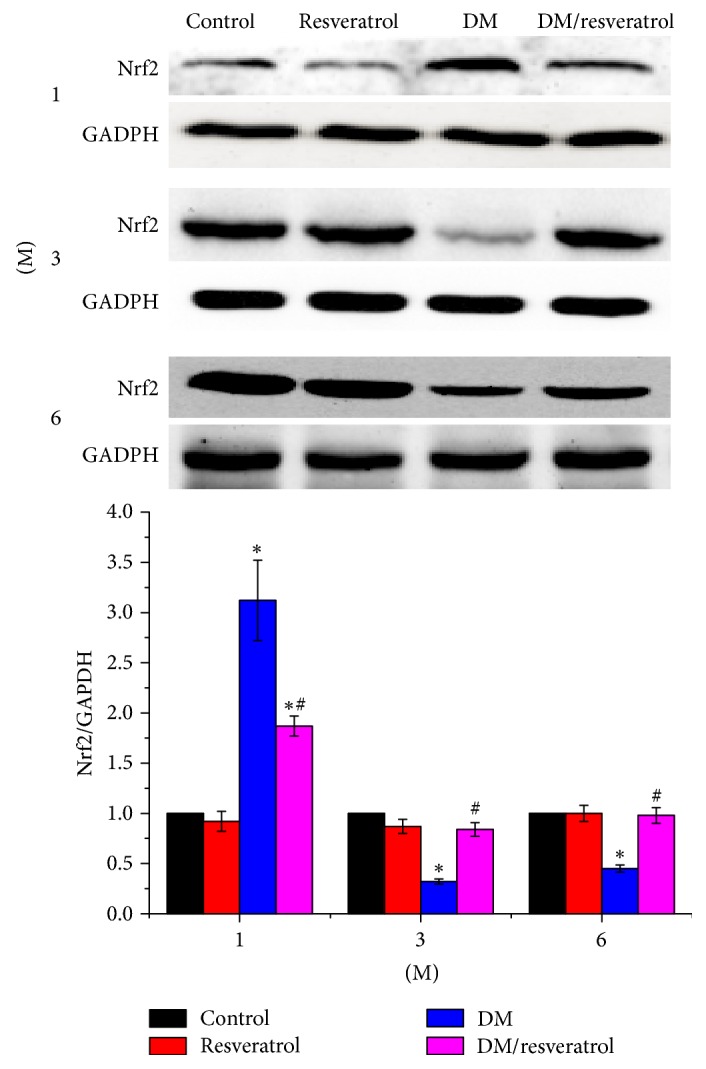
*Resveratrol increases Nrf2 levels in DM mouse hearts. Nrf2 expression was measured by Western blot*. Data are presented as means ± SD (*n* = 6 per group). ^*∗*^*p* < 0.05 versus control; ^#^*p* < 0.05 versus DM group.

**Figure 6:**
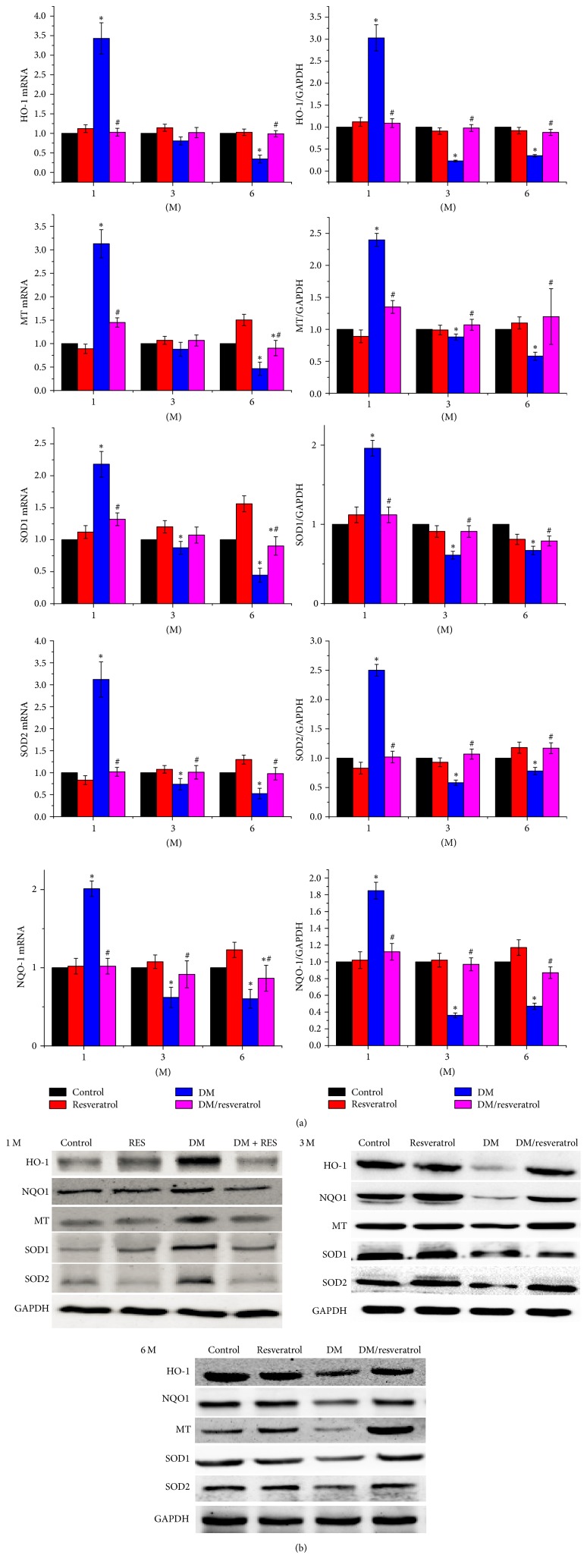
*Resveratrol increases Nrf2 expression and Nrf2's downstream targets in DM mouse hearts.* Expression of Nrf2, as well as Nrf2's downstream genes (SOD1, SOD2, MT, NQO1, and HO-1), was measured by quantitative PCR (a) and Western blot (b). Data are presented as means ± SD (*n* = 6 per group). ^*∗*^*p* < 0.05 versus control; ^#^*p* < 0.05 versus DM group.

The above results suggest that resveratrol attenuates cardiac oxidative damage, fibrosis, and hypertrophy induced by DM. Since resveratrol activates Nrf2 [23], we hypothesized that resveratrol mediates cardiac protection via Nrf2 mediated gene regulation. As shown in Figure 5, Nrf2 expression was significantly increased in the hearts of the DM and DM/resveratrol-treated groups at 1 month after treatment but decreased quickly at 3 and 6 months in the DM group compared to the control and resveratrol groups (^*∗*^*p* < 0.05 versus control). However, resveratrol treatment restored Nrf2 expression in the hearts of the DM group (^#^*p* < 0.05 versus DM). Next, we evaluated Nrf2 transcriptional activity by measuring the levels of its downstream antioxidative gene targets: SOD1, SOD2, NQO1, HQ-1, and MT.  Figure 6 shows that mRNA and protein expression of these targets was increased at 1 month in the DM group, and resveratrol did not alter their expression. At 3 and 6 months, gene expression was reduced in the DM group, and this reduction was completely blocked by resveratrol treatment. Collectively, these findings suggest that resveratrol protects against DM-mediated cardiac injury, at least in part, by increasing Nrf2 expression and transcriptional activity.

## 4. Discussion

In the present study, we evaluated the protective effects and underlying mechanisms of resveratrol in a mouse model of DM-related cardiomyopathy. We found that (1) resveratrol ameliorates cardiac dysfunction and pathological changes including fibrosis and oxidative damage in DM-related cardiomyopathy and (2) Nrf2 plays a significant role in resveratrol-mediated cardiac protection.

In this study, we did not observe any significant difference in blood glucose levels between the DM and DM/resveratrol groups, which was not consistent with previous reports showing that resveratrol reduced hyperglycemia in a STZ model [24–27]. This discrepancy may be attributed to the following: (1) difference in experimental models: the previous studies were performed in a rat experimental model, while we used a mouse model in the present study; (2) differences in dose and duration of resveratrol administration. According to the meta-analysis by Zhu et al. [28], the dose and duration of resveratrol greatly impacted outcomes.

In our study, type 1 diabetes was successfully induced by MLD-STZ injection in mice. This DM mouse model also exhibited DCM, as evidenced by progressive exacerbation of cardiac function, including increased DBP, SBP, and LV mass, decreased LV EF, and increased cardiac fibrosis. Previous studies showed that resveratrol ameliorates cardiac dysfunction in diabetic mice [29, 30]. In line with these reports, we also found that resveratrol attenuates DM-related cardiac dysfunction and pathological changes. Moreover, the increased oxidative stress in DM hearts, as revealed by elevated expression of 3-NT and 4-HNE, was suppressed by resveratrol. Therefore, our data indicate that resveratrol inhibits oxidative stress and preserves cardiac function in DM.

In the present study, we observed that SBP and DBP levels were significantly lower in the DM/resveratrol group compared to the DM group. Previous studies have shown that resveratrol decreases BP by alleviating oxidative stress, increasing endothelial nitric oxide production, and reducing renal inflammation and angiotensin II expression [31–34]. The protective effect of lowering BP on cardiomyocytes has been well established, but we provide further evidence that resveratrol protects against cardiomyocyte injury, at least partially, by directly lowering BP.

Clinically, failure to reverse cardiac pathological changes induced by uncontrolled glucose levels is a limiting factor in treating patients with diabetes. This severe clinical limitation is often referred to as metabolic or hyperglycemic memory. Previous studies have shown that intensive glucose control at an early stage, but not at a late stage, often offers benefits in the prevention of DM-related cardiovascular disorders. Also, effective glucose control achieved only at a late stage of diabetes failed to offer significant benefits to the complication deterrence [35–39]. Therefore, if the early pathogenic changes related to diabetes can be effectively prevented, the likelihood of developing diabetic cardiomyopathy at a late stage will be substantially decreased. Here, we demonstrate that resveratrol treatment protects against-DM-related cardiomyopathy, both acutely (1 month) and chronically (6 months). These findings suggest that resveratrol attenuates DM-related cardiac dysfunction at an early stage, thus providing the experimental basis for the clinical use of resveratrol for preventative treatment of DM-related cardiomyopathy.

Nrf2, a transcription factor, is upregulated in response to oxidative stress [23]. Nrf2 plays an important role in antioxidative responses via multiple mechanisms [40]. Nrf2 expression has been associated with glucose levels in both* in vitro* and* in vivo* studies. For instance, administration of glucose at 20 and 40 mM for 24 h elevated Nrf2 levels in primary cultured cardiomyocytes and in cultured H9c2 cardiac cells [35]. This increase in Nrf2 expression and/or Nrf2's downstream antioxidant genes (NQO1 and HO-1) in response to hyperglycemia was evident in diabetic mouse hearts [35, 41]. Moreover, reduced Nrf2 expression was detected in the hearts of patients with chronic diabetes [42], and Nrf2 expression and activity were involved in cardiac protection in a mouse study of Nrf2-null cardiomyocytes [41]. Consistent with the reported findings, we found that cardiac Nrf2 expression in DM mice significantly decreased at 3 and 6 months, coincident with impaired cardiac function. The decrease in Nrf2 expression in DM mice was attenuated by resveratrol, which also increased the levels of Nrf2's downstream antioxidative targets. Considering the previous finding in light of our current data, we speculate that resveratrol protects cardiac tissue from DM-related damage, at least partially, by modulating Nrf2 expression and function. We noted that DM hearts exhibited increased levels of Nrf2 at 1 month after DM. One previous study also showed that Nrf2 protein levels were marginally increased in the hearts of mice after 2 months of hyperglycemia [42]. The exact mechanism underlying this transient increase in Nrf2 expression in DM hearts remains unknown; however, it is likely that the increased expression of Nrf2 at an early stage of diabetes potentially involves a self-protection mechanism in response to DM-related cardiac injury. This potential mechanism is supported by a previous finding that demonstrated that Nrf2 deletion predisposed cardiomyocytes and hearts to hyperglycemia-induced injury compared to wild-type cells and mice, respectively [41].

Previously, Cheng et al. found that resveratrol significantly potentiated the antioxidant enzymatic activity of GSH-PX and SOD and reduced the expression of MDA in a myocardial ischemia/reperfusion rat model [23]. Resveratrol also upregulated HO-1 and Nrf2 expression. Therefore, they concluded that resveratrol offered significant antioxidant and cardioprotective benefits following myocardial ischemia, at least in part, by activating Nrf2/ARE signaling [23]. In line with this, we also showed that resveratrol enhanced the expression of a number of antioxidative genes in DM-damaged cardiac tissues. However, whether the resveratrol-mediated cardiac protection in DM mice is fully governed by increased expression and activity of cardiac Nrf2 remains unclear and warrants further investigation using a Nrf2-null mouse model [30].

In summary, we demonstrated that resveratrol protects against DCM in a type 1 diabetes mouse model. We also showed that resveratrol promotes Nrf2 expression and function, as well as Nrf2's downstream antioxidative gene targets, in DM-damaged cardiac tissues. Our data suggest that resveratrol exerts protective effects against DM-induced cardiomyopathies by suppressing oxidative stress. Given that resveratrol ameliorates cardiac dysfunction in diabetic mice at an early stage of diabetes, our findings present the experimental basis for potential therapeutic use of resveratrol for acute treatment of DM-related cardiomyopathy.

## Figures and Tables

**Table 1:** Effects of resveratrol on blood pressure and heart rate in unanesthetized DM mice.

Group	1 month			3 months			6 months		
	HR (beats/min)	Diastolic BP (mm Hg)	Systolic BP (mm Hg)	HR (beats/min)	Diastolic BP (mm Hg)	Systolic BP (mm Hg)	HR (beats/min)	Diastolic BP (mm Hg)	Systolic BP (mm Hg)
Control	637 ± 36	74.20 ± 3.27	105.32 ± 2.64	647 ± 35	75.10 ± 3.47	104.12 ± 2.34	640 ± 28	76.10 ± 2.45	106.13 ± 1.92
Resveratrol	645 ± 33	73.22 ± 3.85	102.29 ± 2.55	650 ± 32	74.42 ± 3.81	100.29 ± 2.35	646 ± 19	75.46 ± 2.25	102.16 ± 1.86
DM	646 ± 30	85.93 ± 2.41^*∗*^	115.26 ± 2.23^*∗*^	646 ± 29	86.93 ± 2.61^*∗*^	116.89 ± 2.37^*∗*^	636 ± 16	93.12 ± 3.25^*∗*^	121.75 ± 2.15^*∗*^
DM/resveratrol	636 ± 36	80.60 ± 3.82^#^	104.23 ± 3.00^#^	630 ± 40	79.65 ± 2.62^#^	103.13 ± 3.11^#^	629 ± 20	81.02 ± 1.63^#^	108.13 ± 2.46^#^

Data are presented as means ± SEM. HR = heart rate; BP = blood pressure. ^*∗*^*p* < 0.05 versus control. ^#^*p* < 0.05 versus DM group.

**Table 2:** Effects of resveratrol on cardiac dysfunction and heart rate in anesthetized DM mice (1 month).

Group	HR (beats/min)	LVID (mm)	IVS (mm)	LVPW (mm)	% EF (%)	% FS (%)
Control	496 ± 20	3.60 ± 0.23	0.71 ± 0.05	0.82 ± 0.1	89 ± 5	62 ± 4
Resveratrol	459 ± 24	3.58 ± 0.32	0.72 ± 0.04	0.84 ± 0.15	88 ± 4.0	58 ± 4.5
DM	440 ± 26	3.59 ± 0.32	0.74 ± 0.05	0.86 ± 0.17	78 ± 5.0^*∗*^	49 ± 5^*∗*^
DM/resveratrol	465 ± 24	3.59 ± 0.38	0.73 ± 0.04	0.85 ± 0.12	84 ± 5^#^	55 ± 4^#^

Data are presented as means ± SEM. LVID = LV end-diastolic diameter; LVPW = LV posterior wall; IVS = interventricular septum; FS = fractional shortening; EF = ejection fraction. ^*∗*^*p* < 0.05 versus control. ^#^*p* < 0.05 versus DM group.

**Table 3:** Effects of resveratrol on cardiac dysfunction and heart rate in anesthetized DM mice (3 months).

Group	HR (beats/min)	LVID (mm)	IVS (mm)	LVPW (mm)	% EF (%)	% FS (%)
Control	496 ± 20	3.60 ± 0.13	0.72 ± 0.04	0.83 ± 0.10	90.46 ± 4.69	62.14 ± 4.49
Resveratrol	459 ± 24	3.59 ± 0.12	0.73 ± 0.03	0.86 ± 0.02	88.16 ± 4.64	58.76 ± 4.42
DM	440 ± 26	3.74 ± 0.16	0.75 ± 0.02^*∗*^	1.03 ± 0.04^*∗*^	68.22 ± 4.01^*∗*^	39.22 ± 3.18^*∗*^
DM/resveratrol	465 ± 24	3.62 ± 0.21	0.73 ± 0.01^#^	0.9 ± 0.02^#^	84.18 ± 3.03^#^	49.10 ± 4.61^#^

Data are presented as means ± SEM. LVID = LV end-diastolic diameter; LVPW = LV posterior wall; IVS = interventricular septum; FS = fractional shortening; EF = ejection fraction. ^*∗*^*p* < 0.05 versus control. ^#^*p* < 0.05 versus DM group.

**Table 4:** Effects of resveratrol on cardiac dysfunction and heart rate in anesthetized DM mice (6 months).

Group	HR (beats/min)	LVID (mm)	IVS (mm)	LVPW (mm)	% EF (%)	% FS (%)
Control	483 ± 27	3.6 ± 0.19	0.76 ± 0.04	0.85 ± 0.09	89.35 ± 3.70	60.10 ± 4.86
Resveratrol	478 ± 21	3.58 ± 0.12	0.78 ± 0.02	0.92 ± 0.03	88.24 ± 4.52	56.25 ± 4.73
DM	464 ± 23	3.99 ± 0.12^*∗*^	0.99 ± 0.02^*∗*^	1.05 ± 0.02^*∗*^	66.25 ± 3.25^*∗*^	36.00 ± 3.63^*∗*^
DM/resveratrol	468 ± 17	3.76 ± 0.14^#^	0.83 ± 0.02^#^	0.90 ± 0.02^#^	80.05 ± 3.60^#^	48.22 ± 4.50^#^

Data are presented as means ± SEM. LVID = LV end-diastolic diameter; LVPW = LV posterior wall; IVS = interventricular septum; FS = fractional shortening; EF = ejection fraction. ^*∗*^*p* < 0.05 versus control. ^#^*p* < 0.05 versus DM group.

## Supplementary Materials

Supplementary Materials Resveratrol prevention of diabetes-induced myocardial injury. Photomicrographs of histological hematoxylin-eosin.
