# Supplementary figures and images for: Resveratrol Prevents Diabetic Cardiomyopathy by Increasing Nrf2 Expression and Transcriptional Activity
Source: Biomed Res Int. 2018 Mar 12;2018:2150218. doi: 10.1155/2018/2150218 (PMC5867593; doi:10.1155/2018/2150218)

## Slide 1
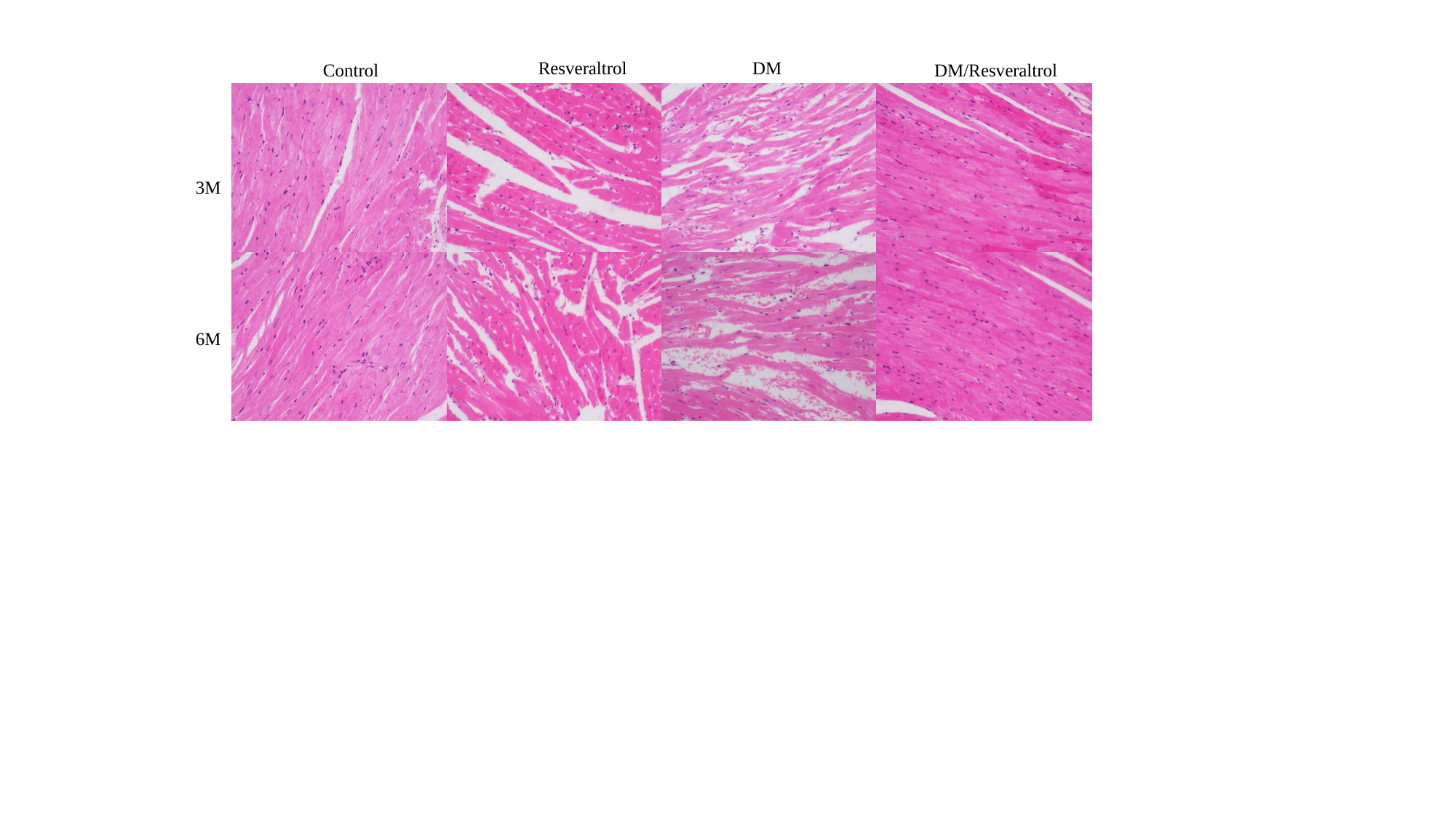

Resveraltrol
DM
Control
DM/Resveraltrol
3M
6M

Supplement: Supplementary Materials — Resveratrol prevention of diabetes-induced myocardial injury. Photomicrographs of histological hematoxylin-eosin. [file 2150218.f1.zip › Supplemental figure .pptx]

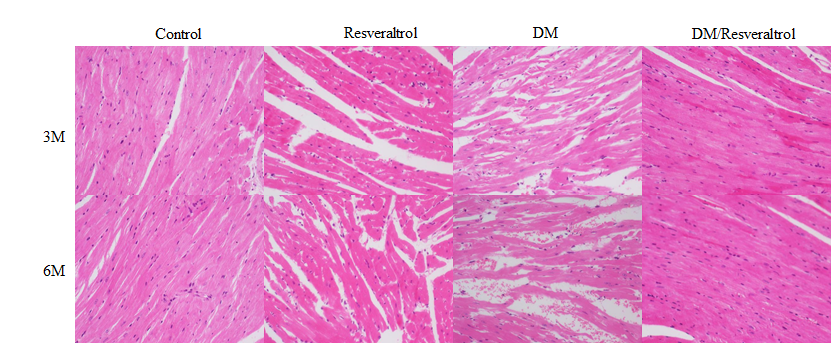

Supplement: Supplementary Materials — Resveratrol prevention of diabetes-induced myocardial injury. Photomicrographs of histological hematoxylin-eosin. [file 2150218.f1.zip › supplementary figure.tif]
